# Exercise, Spinal Microglia and Neuropathic Pain: Potential Molecular Mechanisms

**DOI:** 10.1007/s11064-023-04025-4

**Published:** 2023-09-19

**Authors:** Min-Jia Wang, Xin-Yu Jing, Yao-Zheng Wang, Bi-Ru Yang, Qu Lu, Hao Hu, Liang Kang

**Affiliations:** 1 Institute of Sports Medicine and Health, Chengdu Sports University, Chengdu, 610041 China; 2 Department of Postpartum Rehabilitation, Sichuan Jinxin Women and Children Hospital, Chengdu, 610041 China; 3 School of Sports Medicine and Rehabilitation, Beijing Sport University, Beijing, 100084 China

**Keywords:** Neuropathic pain, Spinal microglia, Exercise, Purine, Fractalkine, p38 MAPK

## Abstract

As one of the most common neuropathic disorders, neuropathic pain often has a negative impact on patients with persistent pain, mood disorders and sleep disturbances. Currently, neuropathic pain is not treated with any specific drug, instead, drugs for other diseases are used as replacements in clinics, but most have adverse effects. In recent years, the role of spinal cord microglia in the pathogenesis of neuropathic pain has been widely recognized, and they are being explored as potential therapeutic targets. Spinal microglia are known to be involved in the pathogenic mechanisms of neuropathic pain through purine signaling, fractalkine signaling, and p38 MAPK signaling. Exercise is a safe and effective treatment, and numerous studies have demonstrated its effectiveness in improving neurological symptoms. Nevertheless, it remains unclear what the exact molecular mechanism is. This review summarized the specific molecular mechanisms of exercise in alleviating neuropathic pain by mediating the activity of spinal microglia and maintaining the phenotypic homeostasis of spinal microglia through purine signaling, fractalkine signaling and p38 MAPK signaling. In addition, it has been proposed that different intensities and types of exercise affect the regulation of the above-mentioned signaling pathways differently, providing a theoretical basis for the improvement of neuropathic pain through exercise.

## Introduction

A major contributor to the global burden of disease, neuropathic pain (NPP) affects between 6.9% and 10% of the general population [1]. As well as persistent pain, neuropathic pain is often accompanied by mood disorders and sleep disturbances, which adversely affect patients’ quality of life [2, 3]. Currently, the primary treatment modalities for NPP are pharmacological. Opioids, cannabinoids, botulinum toxin, topical capsaicin, gabapentin, pregabalin, inhibitors of serotonin and noradrenalin reuptake, and tricyclic antidepressants (TCA) are recommended as first-line treatments according to evidence of high or moderate quality. TCAs inhibit neuronal hyperexcitability mainly by blocking Na^+^ and Ca^2+^ and adenosine activity [4]. However, TCAs can cause side effects such as dry mouth, confusion, urinary retention, and potential cardiotoxicity when taken for long periods of time [5]. The anticonvulsant carbamazepine decreases neuronal Na^+^ and Ca^2+^ inward flow, thereby reducing injurious stimulation of neurons [6], but the anticonvulsant carbamazepine also causes blurred vision, diplopia, nystagmus, and water intoxication when taken for long periods of time [7]. While several of these drugs have been developed for other indications (e.g. depression and epilepsy), there are no drugs that are specifically indicated for treating NPP. Additionally, these drugs have treatment-related adverse effects in the clinical phase. Meanwhile, these clinical drugs exert their therapeutic effects on NPP via Na^+^ and Ca^2+^ ion channels and do not regulate spinal microglia. Spinal cord microglia play a crucial role in the pathogenesis of neuropathic pain. However, the mechanisms underlying spinal microglial activation during neuropathic pain remain incompletely determined [8, 9]. Microglia are the first responders and principal resident immune cells of the central nervous system [10]. A large body of work has demonstrated that microglial activation is the early event that mediates neuroinflammation in neuropathic pain [11, 12]. Several studies have shown that exercise, as a systemic physical activity, can exert neuroprotective effects by strengthening neurogenesis and reducing the inflammatory response [13–15]. Although physical exercise has a neuroprotective effect by inhibiting microglia, fewer articles have investigated the relationship between exercise and spinal microglia in NPP. With the in-depth study of spinal microglia, a few signaling pathways, including fractalkine, purine signaling and others, are thought to be effective in regulating the expression of spinal microglia in NPP. In addition, the present study hypothesised the factors and signalling pathways involved in regulating NPP and generating neuroprotective effects through spinal microglia motility.

### Microglia in Neuropathic Pain

A growing body of evidence indicates that spinal microglia react and undergo a series of changes that directly influence the establishment of neuropathic pain states [16]. After nerve damage, purinergic P2X4 receptors (nonselective cation channels activated by extracellular ATP) are upregulated in spinal microglia in a manner dependent on transcription factors interferon regulatory factor 8 and 5 (IRF8 and IRF5), both of which are expressed in microglia after peripheral nerve injury [17]. The expression of P2X4 receptors on the surface of microglia is regulated at the post-translational level by the CC chemokine receptor, chemotactic cytokine receptor 2. In addition, spinal microglia respond to extracellular stimuli through intracellular signaling pathways such as the mitogen-activated protein kinase, p38, and extracellular signal-regulated protein kinase pathways [18]. Inhibition of the function or expression of these microglial molecules suppresses the aberrant excitability of dorsal horn neurons and causes neuropathic pain [17].

Central nervous system (CNS) microglia are the main immunoreactive cells, which promote the proliferation and differentiation of neuronal cells and have a role in supporting, protecting and nourishing neurons [19–22]. Peripheral nerve injury activates microglia in the dorsal horn, which releases inflammatory mediators and pro-inflammatory mediators that sensitize neurons, leading to neuropathic pain [23, 24]. As a result, spinal microglia promote neuropathic pain by releasing several glial mediators that sensitize spinal neurons [25]. Meanwhile, microglia are regarded as a main source of inflammatory mediators (IFMs). These IFMs include interleukin-1 beta (IL-1β), interleukin-6 (IL-6), tumor necrosis factor-alpha (TNF-α), prostaglandin E2 (PGE2), nitric oxide, nerve growth factor and others. Spinal microglia activate nociceptive neurons in the CNS and peripheral nervous system (PNS), resulting in hypersensitivity to pain [26, 27]. NPP development was delayed by specific ablation of microglia, microglia produce pro-inflammatory cytokines such as IL-1β [28]. Studies have found that elevated IL-1β decreases the efficacy of morphine analgesia [29]. Also, inhibition of IL-1β can induce an anti-inflammatory response, thereby modulating microglial activation and subsequent pain hypersensitivity [28, 30, 31].

Therefore, according to these studies, spinal microglia play a significant role in the pathogenesis of NPP as well as its therapeutic mechanism. In the treatment of pain, it is expected to be a promising therapeutic target.

### Exercise in Neuropathic Pain

Exercise, as a systemic physical activity, could be neuroprotective by strengthening neurogenesis and reducing inflammation. According to the current study, exercise reduces NPP in both animals and humans [32, 33]. It has been proven that spinal neuron excitability can be regulated by exercise training by increasing the synthesis of GAD-65 and GAD-67 in the dorsal horn of the spinal cord, as well as by reducing nerve potential after spinal cord injury and peripheral nerve injury [34]. An animal experiment found that in streptozotocin (STZ) rats, exercise alleviated mechanical hypersensitivity after four to five weeks. Meanwhile, this study also found that the expression of the mammalian target of rapamycin (mTOR) and IL-6 levels in rat dorsal root ganglions decreased. It seems to indicate that exercise regulates NPP through the mTOR pathway [35]. Moreover, the 5-week swim exercise reversed the increased expression of BDNF in DRG and decreased the up-regulation of NGF, normalized the phosphorylation of PLCg-1 on the dorsal side of spinal cord, and reversed astrocyte and microglia hyperactivity in the dorsal horn after nerve lesion [36]. In summary, exercise stimulates endogenous factors and signaling pathways that affect NPP.

### The Mechanisms that Regulate Exercise to Reduce Neuropathic Pain

After exercise training, cytokines are released in the organism and promote increased synthesis of endogenous factors, such as brain-derived neurotrophic factor (BDNF), glutamic acid decarboxylase (GAD), tumor necrosis factor (TNF), interleukin et al. These factors alleviate NPP through relevant signaling pathways, including mTOR, transmembrane chemokine fractalkine, purine signaling, BDNF-tyrosine-protein kinase B (TrkB) and others [34, 35, 37, 38]. Evidence is now available to prove that these signaling pathways play an important role in NPP via spinal microglia. Additionally, exercise prevents microglia from becoming over-activated [39]. It has been shown that the effects of physical exercise on Alzheimer’s disease (AD) and Parkinson’s disease (PD) could reduce inflammatory processes, inhibit over-activation of microglial cells, and induce neuroprotective effects [13]. There is now evidence that exercise mediates microglia activation or maintains microglia phenotypic homeostasis by upregulating anti-inflammatory factor expression and downregulating pro-inflammatory factor expression. The effect of exercise on neurological symptoms may be mediated by microglia.

## Exercise, Spinal Microglia and Neuropathic Pain

### Exercise Plausible Downregulation of the Expression of Purine Signaling to Relieve Neuropathic Pain

The purinergic system is a critical regulatory pathway in the CNS and a basic signaling system that builds microglial behavior in different conditions. By expressing purinergic P2X and P2Y receptors in microglia, extracellular nucleotides (ADO, ATP, ADP, and AMP) play a critical role in neuron-microglia communication [40]. The microglial purinergic receptor plays a crucial role in neuropathic pain. Neuropathic pain was found to be exacerbated by P2X receptors (P2X4R and P2X7R) and P2Y receptors (P2Y6R, P2Y12R, P2Y13R, and P2Y14R). Activation of P2X/Y receptors can change intracellular molecular metabolism, activate related signaling pathways, release damaging factors, increase sensory information transmission, and trigger pain. It has been reported that sensitive P2X4R mediates increased BDNF and p38-MAPK for pain hypersensitivity [41]. According to studies in vivo, nicotinic antagonist treatment of hyperalgesia models up-regulates P2X4R in spinal microglia [41] and neuropathic pain models [42]. Additionally, blocking P2X4R pharmacologically reduces animals’ pain-related behaviors [43]. In a rat model of chronic constriction of the sciatic nerve (CCI), P2X7R expression is confined to microglia, and spinal microglia are activated after nerve injury [44]. BmK I-induced pain was associated with an increase in the expression of P2X7R in the ipsilateral spinal dorsal horn. The activation of microglial cells in the spinal dorsal horn is mediated by P2X7R up-regulation, which contributes to the development of pain [45]. The study shows that inhibition of p38 MAPK signaling suppressed the increase of P2Y6R, P2Y13R, and P2Y14R expression in microglia. A critical role for p38 MAPK signaling in NPP is played by regulating transcription of P2Y6R, P2Y13R, and P2Y14R in spinal microglia following nerve injury [46]. Both P2Y13R and P2Y12R are associated with microglia in the spinal cord that produce inflammatory cytokines, such as TNF-α, IL-1β, and IL-6 [47]. In vitro, P2Y13R and P2Y12R triggered paracrine signaling during inflammation [43, 48]. According to one study, P2Y12 and P2Y13 receptors regulate NPP synergistically. Nerve injuries increase the expression of P2Y13 receptors. The RhoA/ROCK pathway was activated in microglia, causing neuronal excitability and NPP [49]. A study has shown that the expression of P2Y12R mRNA was obviously increased in the spinal cord ipsilateral to the nerve injury and it was highly restricted to ionized-binding calcium adapter molecule 1-positive microglia expression. A P2Y13 receptor in the dorsal horn may also be involved in diabetic neuropathic pain. A vitro study found that co-culturing the N9 microglial line with a glioma cell line could upregulate P2Y14R expression, suggesting that P2Y14R may play a role in microglia-glioma communication [43, 50].

Currently, there are studies based on P2X and P2Y receptors as potential therapeutic targets. Research on the P2X and P2Y receptors as therapeutic targets for NPP is currently a hot area of research. It has been found that using electroacupuncture and receptor inhibitors can alleviate pain [51–53], but the intervention with exercise is rare. Exercise can regulate P2X and P2Y receptors expression and activation. In other diseases, P2X and P2Y receptors expression can be reduced by exercise to alleviate disease symptoms. The increases or decreases in receptor expression are related to the exercise type, intensity, frequency, and duration. A study has shown that moderate-intensity exercise can reduce the expression of P2X7R, thus regulating the relevant pathways to inflammation. However, low- and high-intensity exercise instead increased the expression of P2X7R [54]. Another study has shown that long-term moderate-intensity aerobic training may effectively reduce the occurrence of atherosclerotic thrombotic events by down-regulating the expression of P2Y12R [55]. Recently, P2X4R and P2X7R were speculated to be priming molecules in exercise-induced changes in BDNF, and a protective mechanism of exercise is provided in neurogenic diseases (including NPP). ATP is released from NPP-stimulated neurons and activates P2X4R and P2X7R in microglia via calcium channels. As purinoceptors become active, the p38 MAPK pathway is triggered, which releases TNF-α, IL-1β and BDNF [56]. Therefore, the regulation of P2X and P2Y receptor downregulation by exercise to alleviate NPP has become a meaningful research area. Choosing an appropriate exercise program is very important.

### Exercise Plays a Double-Sided Role in Affecting Neuropathic Pain by Modulating Fractalkine on Spinal Microglia

C-X3-C motif chemokine ligand 1(CX3CL1, also known as fractalkine), a unique chemokine expressed by neurons constitutively, is a transmembrane chemokine that binds to the membrane. A receptor specific to fractalkine, CX3CR1(the receptor of CX3CL1), is mostly present on the surface of microglial cells within the dorsal spinal cord [57]. There is growing evidence that in the dorsal horn, fractalkine regulates neuron-microglia communication.

As a potential substrate for lysosomal cysteine protease Cathepsin S (CatS) cleavage, fractalkine plays a major role in CatS induction [58]. The CatS protein is essential for the activation of spinal microglia and NPP. The cysteine protease CatS from spinal microglial cells has previously been demonstrated to contribute to maintaining neuropathic hypersensitivity and activating microglia [58]. As we all know, NPP is promoted by the activation of spinal microglia. Thus, the activation of CatS has an induction role for NPP. Conversely, in the dorsal horn, the CatS inhibitor (morpholinurea-leucine-homophenylalaninevinylphenylsulfone, LHVS) attenuates microglia activation and NPP’s analgesic effect is reduced [58]. Research has demonstrated that neuronal soluble fractalkine (sFKN) is released by CatS pronociceptive effects and by CatS-induced microglia activation [25]. As a result of nerve injury, primary afferent fibers are activated, and mediators such as ATP are released, which causes the release of CatS from activated microglia, leading to the rapid release of sFKN. sFKN induces an increase in CX3CR1 that correlates with increased CX3CR1 expression on microglia due to nerve injury, and both together promote injury receptor hypersensitivity. A significant reduction in noxious stimulus responses could be caused by the loss of CX3CR1 [25]. In summary, in neuropathic conditions, increased primary afferent inputs to the dorsal horn cause microglial CatS release, which causes fractalkine liberation from dorsal horn neurons, which contributes to chronic pain amplification and maintenance through activation of CX3CR1 receptors on microglia. When fractalkine is activated on spinal microglia, p38 MAPK-mediated pathways are also activated [25, 58–60]. Due to the strong neuronal excitation in the spinal cord induced by upregulation of CX3CL1 during pathological pain conditions, the receptor expression of fractalkine is increased in microglia in pain-relevant areas [60]. Soluble secreted forms of fractalkine combined with CX3CR1 activate the proliferation and migration of microglia surrounding the affected area [25, 61]. Microglia contribute to enhanced nociceptive signaling when it comes to damaging stimuli to the peripheral nervous system [62]. Evidence shows that after PNI, microglia activation is induced by fractalkine signaling via CX3CR1. In response to p38 activation, proinflammatory cytokines such as TNF-a and IL-1 are synthesized [27]. Fractalkine signaling in the spine activates p38 phosphorylation, and there is a predominant expression of this process in spinal microglia in naive rats. After peripheral nerve injury, intra-cisternal administration of neutralizing antibodies against CX3CR1 could inhibit p38 phosphorylation. The NPP study shows the interaction between fractalkine and its microglial receptor (CX3CR1, P2X7R), which is critically involved in the maintenance of pain [60]. Interestingly, in contrast to its pro-injury effect in NPP, it has been shown that the neuronal fractalkine/microglial CX3CR1 system has a neuroprotective effect in diseases associated with neurodegeneration [63].

Consequently, inhibition of the fractalkine/CX3CR1 pathway may be an effective strategy for alleviating NPP. However, there is variability in the modulatory effects of different exercises on fractaline. Fractalkine expression was significantly upregulated in resistance and acute exercise. A previous study found that the fractalkine mRNA of eight untrained men was significantly elevated 2 h post-intense resistance exercise [64]. Meanwhile, a clinical study found that 12 cytokines and myokines showed significant alterations after low-intensity resistance exercise compared to before exercise. It was found that Fractalkine/CX3CL1 was the most significantly upregulated by exercise [65]. In addition, recent studies have demonstrated that CX3CR1 expression is induced in the spinal cord by this substance during intense acute swimming [66]. However, in a study of aerobic exercise, researchers found that aerobic exercise reduces plasma levels of fractalkine in younger adults [67]. The results of these studies suggest that exercise may regulate fractalin in response to inflammation. Acute exercise and resistance exercise trigger inflammation, while regular aerobic exercise suppresses inflammation [68]. As a result, different exercise intensities and types may have different effects on the expression of NPP.

As a result, we suggested that exercise regulates the fractalkine pathway through spinal microglia. The expression of fractalkine is associated with the pathogenic mechanism of NPP. In this respect, our campaign may suggest that improving NPP via fractalkine is an issue that should be explored further.

### Exercise Downregulates p38 MAPK Signaling, Reducing the Activation of Spinal Microglia and the Generation of Hyperalgesia via Spinal Microglia

Mitogen-activated protein kinases (MAPKs) are a family of evolutionarily conserved molecules that play an important role in cell signaling and gene expression. There are three major members of the MAPK family, representing three different signaling cascades that are known to participate in the generation of hyperalgesia. These members include extracellular signal-regulated kinase (ERK), p38, and c-Jun N-terminal kinase (JNK) [69, 70]. The p38 MAPK is regarded as a stress-induced kinase, and it plays a critical role in inflammation. The inhibitor of p38 has been shown to effectively relieve rheumatoid arthritis and inflammatory pain [71].

Numerous studies have demonstrated that p38 activation in spinal microglia plays a significant role in NPP. The antibody of phosphorylated p38 was used in a study to examine activation of p38 in the rat spinal cord after spinal nerve ligation (SNL), a model commonly used to study NPP. This result indicated that the increase in phosphorylation of p38 is accompanied by an increase in p38 activity, which means ATF-2, a substrate of p38, is phosphorylated at a higher level. Meanwhile, p38 is activated in spinal cells labeled with the microglial marker CD11b [26, 27, 71, 72]. Additionally, recent studies have shown that the application of p38 inhibitors such as FR167653, CNI-1493 and SB203580 can reverse early NPP [27, 71].

There is no doubt that p38 is an important signaling factor for microglia. In recent years, several studies have investigated the upstream mechanisms that cause the activation of p38 by spinal microglia in NPP [9, 71, 73]. It was proven that NPP is associated with p38 MAPK activation and the accumulation of inflammatory cytokines in the spinal cord. As mentioned above, several inflammatory factors, such as IL-1β, IL-6 and TNF-α, are released by nerve spontaneous activity after nerve injury. It is currently confirmed that TNF-α inhibitors prevent NPP development by inhibiting spinal p38 activation [72]. Meanwhile, the hyperalgesia, which is produced by IL-1β and substance P, could be abolished by a p38 inhibitor [74, 75]. Additionally, according to recent research, chemokines have been shown to be important mediators of neural-microglial interactions and the sensitization of NPP [60]. Fractalkine/CX3CR1, a typical chemokine, is activated by CatS on spinal cord microglia following nerve injury, thereby enhancing NPP [58]. Therefore, the development of NPP requires the fractalkine-CX3CR1-p38 cascade. The purinergic system has also been shown to activate p38 by activating receptors on the spinal microglia in NPP. With the continuous peripheral noxious stimulation, maybe the microglial P2X7R is responsible for the release of the sensory neuronal fractalkine, which stimulates the phosphorylation of p38 MAPK in microglia, thereby activating neurons through the release of pronociceptive mediators [76, 77].

Thus, p38 MAPK in spinal microglia is activated by the above 3 pathways. In the downstream mechanism of p38 MAPK, p38 activates phospholipase A2 (PLA2) through its downstream kinase MAPK-activated protein kinase-2 (MAPKAP-2), and after activating PLA2, arachidonic acid is produced for the generation of prostaglandins, which are then catalyzed by COX to produce PGE2 [78]. As previously mentioned, posttranslational regulation by P38 can also control the synthesis of IFMs. Activation of p38 increases the expression of inflammatory mediators and growth factors (including cytokines and BDNF) which are secreted via the transcription factor NF-κB. With the release of these mediators, nociceptive dorsal horn neurons are sensitized via both presynaptic and postsynaptic mechanisms, which leads to persistent hyperalgesia [26].

While there is no clear evidence that exercise can improve NPP through p38 MAPK, research has confirmed that exercise can have neuroprotective effects through p38 MAPK [39, 79]. Hyperalgesia and the decrease of the pain threshold for nociceptive stimulation are the most common manifestations of NPP. P38 phosphorylation plays a key role in the formation of hyperalgesia and tolerance by releasing a multitude of inflammatory mediators from the microglia, including IL-1β, IL-6, and TNF-α [27].

An experiment conducted on mice with morphine-induced hyperalgesia found that running at a speed of 20 m per minute for 30 min every day reduced the development of morphine hyperalgesia and tolerance, and exercise reduced p-p38 expression in the presence of morphine. This is consistent with the results of applying p38 inhibitors (SB203580) [39]. Additionally, this study also used the same exercise protocol to exercise precondition rats after simulated neonatal incision surgery-induced enhanced hyperalgesia and also found that exercise preconditioning reduced hyperalgesia [79]*.* It has been reported that exercise mediates inflammation in the central nervous system, prevents microglia activation, and has antinociceptive effects [39]. As a result of reduced inflammation and inhibition of microglial over activity, p38 phosphorylation is reduced, resulting in an increase in nociceptive thresholds and an improvement in hypersensitivity. There is now evidence that exercise can reduce nociceptive hypersensitivity via the p38 MAPK pathway, while p38 MAPK plays a key role in NPP. Therefore, whether the mechanism by which exercise improves NPP is related to the p38 MAPK pathway is an issue worth exploring in the future.

## Conclusion

It is proven that purine signaling, fractalkine signaling, and p38 MAPK signaling play a significant role in pathological processes affecting NPP via spinal microglia (Table 1). Exercise modulates the p38 MAPK pathway to improve hyperalgesia and produce neuroprotective effects. However, there is limited efficacy in clinical trials based on the current research. Fractalkine and purine are able to affect the NPP, and exercise modulates their expression through spinal microglia. With this evidence it can be hypothesised that exercise can affect the development of NPP by regulating spinal microglia. In addition, the latest in vivo experiment found that exercise enhanced autophagy through the BDNF/ AKT/mTOR pathway and promoted m1-m2 polarization of microglia to improve neuropathic pain. Again, this implies that exercise may improve the path. Furthermore, different intensities and types of exercise, such as moderate-intensity exercise, acute exercise, resistance exercise, and aerobic exercise contribute to the down-regulation of purine receptors, the p38 MAPK pathway, and the simultaneous upward and downregulation of fractalkine. Considering these findings, we speculate that more clinical studies would be necessary to clarify the relationship between the type and intensity of exercise and NPP, as well as its underlying mechanisms in the future.

**Table 1:** Effect of signaling pathways on Neuropathic pain via spinal microglia

Signaling	Mediator	Effects of spinal microglia	Effects of neuropathic pain	Reference
Purine signaling	( +) P2X ↑P2X4R and P2X7R ( +) P2Y ↑P2Y6R, P2Y12R, P2Y13R, and P2Y14R	( +) Microglia in the spinal dorsal horn ( +) FKN ( +) p38 MAPK	Exacerbating neuropathic pain	[39–55]
FKN signaling	↑CatS and CX3CL1 ( +) CX3CR1	( +) Spinal microglia ( +) p38 MAPK	Promoting neuropathic pain	[57–67]
P38 MAPK signaling	( +) PLA2; ↑PGE2; ( +) NF-kB ↑IL-1 β, IL-6, TNF- α and BDNF	( +) Spinal microglia	Inducing the hyperalgesia and the decreasing of pain threshold for nociceptive stimulation	[71–78]

+ : be activated; ↑: upregulation

## Data Availability

Not applicable.

## Abbreviations

AD: Alzheimer’s disease
BDNF: Brain-derived neurotrophic factor
CatS: Cathepsin S
CCI: A rat model of chronic constriction of the sciatic nerve
CNS: Central nervous system
CX3CL1/fractalkine: C-X3-C motif chemokine ligand 1
ERK: Extracellular signal regulated kinase
GAD: Glutamic acid decarboxylase
IFMs: Inflammatory mediators
IL-1β: Interleukin-1 beta
IL-6: Interleukin-6
JNK: C-Jun N-terminal kinase
LHVS: Morpholinurea-leucine-homophenylalanine-vinylsulfone phenyl
MAPK: Mitogen-activated protein kinases
MAPKAP-2: MAPK activated protein kinase-2
mTOR: Mammalian target of rapamycin
NPP: Neuropathic pain
PD: Parkinson’s disease
PGE2: Prostaglandin E2
PNS: Peripheral nervous system
sFKN: Soluble fractalkine
SNL: Spinal nerve ligation
STZ: Streptozotocin
TCA: Tricyclic antidepressants
TNF: Tumor necrosis factor
TNF-α: Tumor necrosis factor-alpha
TrkB: BDNF-tyrosine-protein kinase B
PLA2: Phospholipase A2
